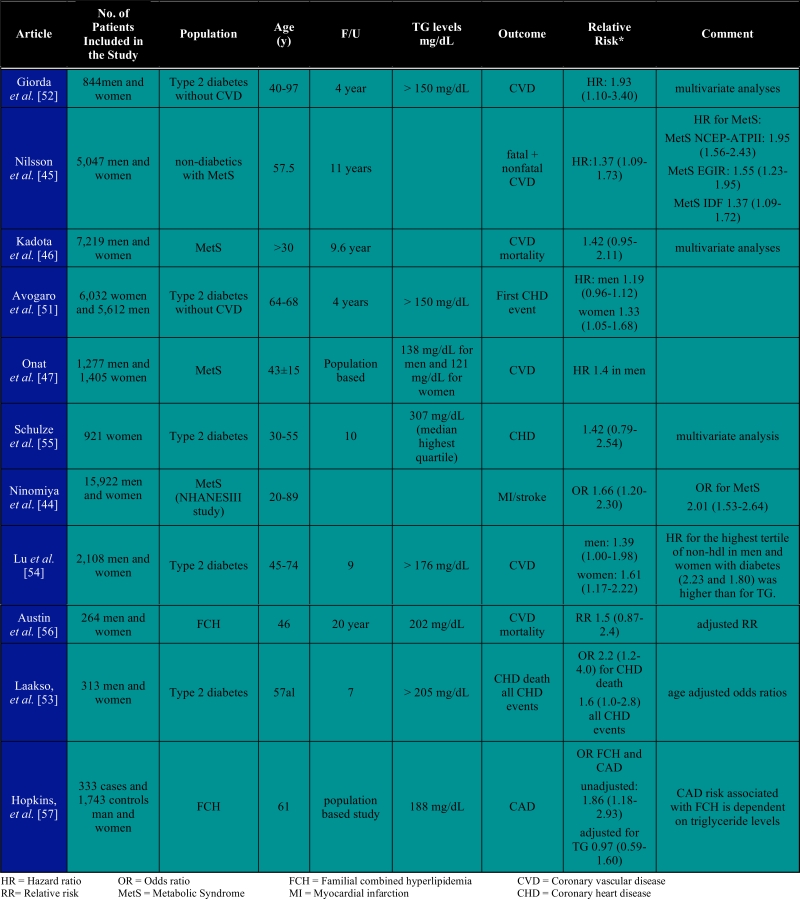# Triglycerides and Cardiovascular Risk

**DOI:** 10.2174/157340309788970315

**Published:** 2009-08

**Authors:** K.E.L Harchaoui, M.E Visser, J.J.P Kastelein, E.S Stroes, G.M Dallinga-Thie

**Affiliations:** 1 Department of Vascular Medicine, Academic Medical Center Amsterdam, The Netherlands; 2 Laboratory of Experimental Vascular Medicine, Academic Medical Center Amsterdam, The Netherlands

**Keywords:** Triglycerides, coronary heart disease, lipoproteins, lipolytic enzymes.

## Abstract

In 1996 a meta-analysis was published showing that an increase in plasma triglyceride (TG) levels was associated with an increase in CHD risk, even after adjustment for high density lipoprotein cholesterol (HDL-C) levels. Very recently, two studies were published that further extent the early observation and showed the importance of nonfasting plasma triglyceride (TG) levels in the prediction of risk on coronary heart disease (CHD). In the current review we have summarized all available evidence obtained in clinical studies showing that treatment guidelines should reconsider to include nonfasting TG in their risk assessments as nonfasting TG levels may better predict CVD risk.

## INTRODUCTION

Despite the introduction of a plethora of pharmacological and other modalities, cardiovascular disease (CVD) is still a principal cause of morbidity and mortality in Western society [1]. The use of HMG-CoA inhibitors (statins) have led to impressive reductions in low-density lipoprotein cholesterol levels (LDL-C). However, only moderate reductions in total mortality were achieved. As a result, current therapeutic guidelines advocate a more stringent LDL-C target in patients at very high or high risk. Since the majority of patients who are currently seen at out-patient clinics display a different lipid phenotype than in the past, the guideline committees have added non-high density Lipoprotein cholesterol (non-HDL-C) as a secondary goal for those with TG levels above 200 mg/dL (1.5 mmol/L) [2-4]. Worldwide, the general population is becoming more obese. This leads to an increase in the prevalence of the so-called atherogenic lipoprotein profile with increased levels of non-HDL-C, apolipoprotein (apo)B, TG as well as decreased levels of HDL-C. Moreover, an increased prevalence of small dense LDL particles is observed. The purpose of the current review is to assess all evidence showing that both TG and non-HDL-C, a marker reflecting both atherogenic LDL and very low density lipoprotein (VLDL) particles, are independent risk factors for CVD.

## TRIGLYCERIDE METABOLISM (FIG. 1)

Plasma TG are derived from dietary sources as well as from de novo TG synthesis [5]. During its path through the digestive system, dietary fat is readily lipolysed, dissolved into micelles and finally hydrolysed by pancreatic lipase, thereby enabling the uptake of fatty acids in the small intestine [6]. Upon its entry into the enterocyte fatty acids will be converted into TG by the enzyme acyl-CoA:diacylglycerol acyltransferase 2 (DGAT2)[7]. TG can be stored in lipid droplets but most of the TG will be packed in apoB-48-containing lipoprotein particles (chylomicrons) by microsomal triglyceride transfer protein (MTTP) and subsequently secreted in the lymphatic system which directly drains into the systemic circulation bypassing the liver [8]. 

De novo lipogenesis occurs in the liver. Released fatty acids from adipose tissue, the TG storage pool in the human body, are directly taken up by the liver and can be considered as major source for substrate. The liver exclusively synthesizes apoB-100 containing VLDL particles. There are large similarities between the processing of apoB-48-containing chylomicron particles in the enterocytes and apoB-100-containing VLDL particles in the liver involving DGATs and MTP. The liver synthesizes a TG-poor VLDL particle (VLDL_2_) [9]. VLDL_2_ can either be secreted by the hepatocyte or further lipidated to form a mature, triglyceride-rich VLDL (VLDL_1_) [10]. VLDL assembly is dependent on the accumulation of TG in the liver, and it has been suggested that the fatty acids used for the biosynthesis of VLDL-TG are derived predominantly from TG stored in cytosolic lipid droplets [10].

Upon secretion, TGs are directly lipolysed by lipoprotein lipase (LPL) present in the capillary beds of adipose tissue and skeletal muscle. ApoC-II acts as an important co-factor. Of note, patients deficient in apoC-II have severe hypertriglyceridemia [11]. A number of different proteins such as apoC-III, apoA-V, angiopoietin-like proteins ANGPTL 3 and 4 are also present in the circulation and may act as potential inhibitors for the action of LpL [12]. The released fatty acids can subsequently be taken up by the adipose tissue for energy storage and by skeletal muscle where they can be directly used for energy. In the heart, LPL-derived fatty acids are responsible for up to 70% of the energy source [13]. The remaining apoB-48 containing chylomicron remnants will be directly taken up by the liver through a concerted action of heparin sulphate proteoglycans (HSPG), which can rapidly bind apoB-48, LDL receptor-related protein 1 (LRP1) and by the LDL-receptor located on the plasma membrane that will bind apoE present on the chylomicron remnant particle [7, 14, 15]. Further modulation of apoB-100-containing VLDL remnant particles, mediated by the action of LpL, hepatic lipase (HL) and cholesteryl ester transfer protein (CETP), will eventually lead to the formation of a cholesterol-enriched LDL particle that is finally cleared by hepatic LDL receptors. TGs are water-insoluble and are thus located in the core of the particle. A decrease in the TG content of the particle leads to shrinking of the particle and subsequently abundant phospholipid-rich surface material will contribute to the formation of lipid-poor apoA-I enriched pre-β HDL particles, mediated by phospholipid transfer protein (PLTP) [16]. CETP and PLTP activities will increase in the presence of an increased pool of TG-rich lipoprotein particles and may therefore contribute to decreased HDL-C levels and an increased concentration of atherogenic small dense LDL in hypertriglyceridemic conditions [17].

Hypertriglyceridemia is often observed in patients with the metabolic syndrome (MetS), type 2 diabetes (DM) or familial combined hyperlipidemia (FCHL) [18, 19]. The hallmark of these clinical phenotypes is the presence of insulin resistance [10]. Insulin plays an important role in the regulation of lipid homeostasis. In the insulin resistant state, the normal response to insulin is attenuated. This will eventually lead to abnormalities in lipid handling in those organs that are particularly sensitive to insulin regulation such as adipose tissue, liver and skeletal muscle. In the fed state, insulin activates LPL in adipose tissue and thereby set the switch to uptake of free fatty acids (FFA) into the adipocytes which will then be stored in lipid droplets. In the fasted state, when insulin levels are low, glucagon will upregulate hormone sensitive lipase (HSL) by inactivation of cAMP mediated PKA-dependent phosphorylation of HSL thereby enabling the formation of FFA which are secreted and taken up by peripheral tissues [20]. Adipose tissue triglyceride lipase (ATGL), an enzyme that is involved in the first step of TG hydrolysis may act independent of hormonal activation [20]. In the liver, increased FA supply, due to adipose tissue insulin resistance, leads to abnormal VLDL synthesis and secretion through the enhanced TG pool in the liver [10]. It has since long been recognized that fatty acids are important ligands for several nuclear receptors that control lipid metabolism [21]. Abnormal signalling, as observed in the insulin resistant state, therefore contributes extensively to the up-regulation in hepatic lipoprotein assembly seen in hypertriglyceridemia. 

## TG AND CVD RISK

Hokanson and Austin already showed in 1996 in a meta analysis that TG are an independent risk factor for CVD [22]. Presence of elevated concentrations of atherogenic TG-rich remnant lipoproteins is the principle abnormality responsible for this association [23]. However, the evidence for TG being an independent risk factor for CHD remains to some extent controversial because data obtained in large clinical studies were not always equivocal. Moreover, in the case of positive findings, the effect sizes were very modest. A number of issues have been raised to explain these inconclu-sive results. Plasma TG are a highly variable lipid compo-nent, as it reflects the daily consumption of dietary fat. Nowadays humans consume at least three consecutive meals per day. As a consequence, people are in a continuous post-prandial state which is reflected in highly variable plasma TG levels [24]. Secondly, patients with high TG levels frequently exhibit additional risk factors, such as insulin resistance that may affect their susceptibility to athero-sclerosis. Finally, elevated plasma TG levels are strongly associated with low HDL-C levels [25]. In prospective epidemiological studies it will thus be difficult to separate the contributions of HDL and TG to atherosclerosis suscep-tibility. Based on these limitations, investigators have argued that maybe non-HDL-C would be a better marker to explain the whole spectrum of atherogenic lipoprotein particles including LDL, small dense LDL and VLDL remnants. Indeed a recent post hoc analysis was performed using combined data of the ‘Treating to New Targets’-TNT trial and the ‘Incremental Decrease in End Points through Aggressive Lipid Lowering’-IDEAL trial and it revealed that on-treatment levels of non-HDL-C but also apoB were more closely associated with cardiovascular outcome than LDL-C or total cholesterol levels [26]. 

A number of population studies have investigated whether plasma TG are an independent risk factor for CVD. The Prospective Cardiovascular Münster (PROCAM) study involved 4849 middle-aged men who were followed up for 8 years to record the incidence of coronary heart disease (CHD) events according to the risk factors present at study entry. The study showed that fasting levels of TG were an independent risk factor for CHD events, independent of serum levels of HDL-C or LDL-C [27]. In the 8-year Copenhagen Male Study [28], a prospective study including 2906 men who were free of cardiovascular disease at baseline, the risk for ischemic heart disease was 50% higher in those with TG levels in the middle tertile and 120% higher in the upper tertile as compared to those in the lowest TG tertile after adjustment for conventional risk factors such as age, body mass index (BMI), alcohol intake, smoking, physical activity, hypertension, diabetes, and LDL-C and HDL-C levels. A number of meta-analyses have been published showing independent associations between TG levels and CHD risk, independent of HDL-C levels [22, 29, 30]. A relative risk of 1.32 (95%CI 1.26-1.39) in men and 1.76 (95% CI 1.50-2.07) in women was originally found [31]. More recent analyses in individuals selected from the Reykjavik cohort and the Epic-Norfolk cohort revealed an odds ratio for CHD of 1.76 in the former and 1.57 in the latter when comparing individuals in the lowest tertile for TG as compared to those in the highest tertile, which was similar to data derived from a meta-analysis [30]. In line with these results are the data derived in a study of 13,953 young men for which Tirosh *et al. *[32] reported on an association between the first of 2 TG measurements obtained 5 years apart with incident CHD. After an average follow-up of 10.5 years, men in the fifth quintile of TG, compared with those in the lowest quintile, had a multivariable hazard ratio for CHD of 4.1. Additionally, it has been reported that in patients with CHD TG levels were significantly related to secondary CVD events independent of HDL-C and LDL-C with a HR of 1.50 [33].

Recently, the debate has arisen focused on fasting versus non-fasting plasma TG. Two publications showed a strong association between non-fasting plasma TG and CVD risk [24, 34-36]. In the Copenhagen City Heart Study which included 19,329 men and women with an average follow up of 26 years, nonfasting TG levels were associated with increased risk of myocardial infarction, ischemic heart disease and death. An expansion of this study was recently published where they studied 33,391 inhabitants of Copenhagen (Denmark) [36]. In the Women’s Health Study, which comprised 26,509 women, nonfasting TG levels were associated with incident cardiovascular events, independent of traditional cardiac risk factors, levels of other lipids, and markers of insulin resistance. Recently, data obtained from a prospective study in 26,330 healthy women, revealed that the association with CVD was stronger for nonfasting TG compared with fasting measurements of TG [24]. All these observations combined suggest that nonfasting TG measurements reflect more accurately the presence of atherogenic remnant lipoproteins.

As mentioned above, elevated plasma TG levels are frequently observed in conditions which share a similar metabolic background, such as type 2 diabetes [18], the metabolic syndrome [37] and familial combined hyperlipidemia FCHL [38]. A central hallmark is the presence of insulin resistance. Such patients share the presence of the atherogenic lipoprotein phenotype being increased plasma TG, decreased HDL-C and increased prevalence of small dense LDL particles. Thus the combination of increased hepatic VLDL secretion, and impaired LPL-mediated TG clearance in combination with abnormal function of lipid transfer proteins explains the hypertriglyceridemia phenotype. Consequently, central insulin resistance is the underlying pathophysiological abnormality contributing to peripheral hypertriglyceridemia. In the next paragraphs we will summarize the available evidence that plasma TG are associated with CVD risk in these conditions. As a basis, we have used a pubmed search based on the keywords: TG, metabolic syndrome (MetS), familial combined hyperlipidemia (FCHL), type 2 diabetes (DM), and CVD risk. Moreover, we only used those articles published within the last 10 years. In total we have selected 11 articles that met our inclusion criteria, of which one was published in 1993 and the others were published in 2000 or later (Table**1**).

## TG AND CVD RISK IN TYPE 2 DIABETES, METABOLIC SYNDROME AND FAMILIAL COMBINED HYPERLIPIDEMIA (TABLE 1)

MetS is defined as the presence of a cluster of risk factors including: waist circumference > 102 cm in men or > 88 cm in women, fasting serum TG > 150 mg/dL; blood pressure > 130/85mmHg; HDL < 40 mg/dL in men or < 50 mg/dL in women and fasting serum glucose > 110 mg/dL [39-41]. Individuals that have at least three out of the five criteria are diagnosed as having MetS. As a consequence MetS population studies have to deal with a large heterogeneity which may lead to underestimation of the risk factor estimation. Yet, a large number of population studies have been published using the above described inclusion criteria. Several studies have shown that presence of the MetS or diabetes is associated with increased CVD risk. In the European DECODE (Diabetes Epidemiology: Collaborative Analysis Of Diagnostic Criteria in Europe) study, non-diabetics with MetS had an increased risk of all cause mortality (men: 1.4; women: 1.38) as well as from CVD (men: 2.26; women: 2.78) [42]. In a prospective cohort study, Lakka *et al. *found that men with the metabolic syndrome were at greater risk of cardiovascular disease (CVD) and all-cause mortality, even in the absence of baseline CVD and diabetes [43]. The question whether plasma TG may be considered as an independent risk factor for the prediction of CVD is only addressed in a small number of studies as summarized in Table **1**. Because MetS is not a single entity but consists of a cluster of cardiovascular risk factors it is difficult to show whether TG are independently associated with CVD in these studies. Studies that included analyses of prediction of CVD based on the MetS and TG showed comparable risk ratio’s for MetS and TG [44-47]. Thus, recognition of the syndrome is important because people with metabolic syndrome are more likely to die from coronary heart disease or stroke compared with people without the syndrome [48]. More importantly patients with MetS are prone to develop type 2 diabetes and subsequently CVD [49].

Patients with diabetes have lower HDL-C and higher TG concentrations. Data from the Framingham Heart Study [50] show that twice as many diabetic than non-diabetic individuals have low plasma levels of HDL-C and elevated TG The strong inverse relationship between TG and HDL-C hampers the assessment of the attributable risk of TG and CVD in diabetes. In patients with diabetes mellitus plasma TG concentration was significantly associated with CVD in univariate analyses in several studies [51-55]. However, the independent association of TG with CVD disappeared in most studies when other lipid risk factors, especially HDL-C levels, were taken into account. Interestingly non-HDL-C appears to be a better predictor of CVD risk in diabetes in the presence of high TG levels [54, 55]. Only two studies were published addressing the question whether plasma TG is a major independent risk factor in FCHL [56, 57]. The observed relative risk was similar to that of type 2 diabetes and MetS.

## CONCLUSIONS

Epidemiological evidence indicates that plasma TG levels predict CVD. The independent relationship between TG and CHD is more consistently observed in the general population. In patients with the MetS or diabetes, who share a common metabolic background, plasma TG appear as independent predictors of CVD risk, but the data are inconsistent. The strong inverse relationship between TG and HDL-C in these patients may reflect a common pathophysiologic process and hampers the statistical analyses. For CVD risk estimation and subsequently therapeutic interventions it is therefore recommended to take into account both high TG and low HDL-C levels. Accumulating evidence shows that nonfasting TG levels may better predict CVD risk. Treatment guidelines should reconsider to include nonfasting TG in their risk assessments.

## Figures and Tables

**Fig. (1). Lipoprotein metabolism in the insulin resistance state.**
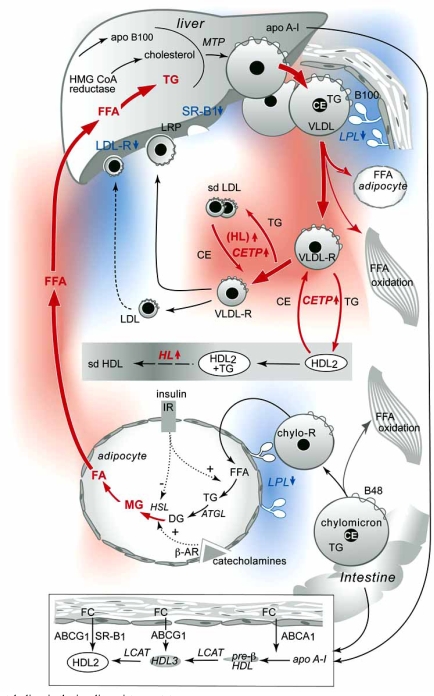
In the insulin resistance state (IR) an enhanced lipolysis in adipocytes, due to increased HSL activity while ATGL activity is normal, results in an increased supply of FFA to the liver which will subsequently lead to increased hepatic TG storage. As a consequence VLDL production and secretion is increased and LPL activity may be reduced. Increased amounts of TG-rich lipoproteins result in an enhanced formation of small dense LDL particles, due to enhanced activity of CETP mediated exchange of cholesteryl esters and TG between HDL and TG-rich lipoproteins. TG-enriched HDL particles may be modulated by hepatic lipase (HL) resulting in smaller–sized HDL_3_ particles which will be rapidly cleared and explains the decrease in plasma HDL-C. Scavenger receptor B1 (SR-B1) may be involved in the hepatic clearance of HDL-cholesteryl esters. There may be a delay in clearance of LDL particles due to decreased expression of the LDL receptor. **Abbreviations:** IR insulin resistance; apo, apolipoprotein; FFA, free fatty acids; CETP cholesteryl ester transfer protein, HL hepatic lipase; ATGL adipose tissue triglyceride lipase; HSL hormone sensitive lipase; MTP microsomal triglyceride transfer protein; SR-B1 scavenger receptor B1. This figure is adopted from Westerveld H.E. *et al.* [58] and published with permission of Elsevier.

**Table 1:** TG as an Independent Marker for Atherosclerosis in Patients with Metabolic Syndrome, type 2 Diabetes and Familial Combined Hyperlipidemia. Articles are Presented by Publication Date, Starting with the most Recent Published Data